# Demodex folliculorum infestations in common facial dermatoses: acne vulgaris, rosacea, seborrheic dermatitis^☆^^☆☆^

**DOI:** 10.1016/j.abd.2019.08.023

**Published:** 2020-02-12

**Authors:** Ezgi Aktaş Karabay, Aslı Aksu Çerman

**Affiliations:** Department of Dermatology and Venereology, Faculty of Medicine, Bahçeşehir University, Istanbul, Turkey

**Keywords:** Acne vulgaris, Dermatitis, seborrheic, Rosacea

## Abstract

**Background:**

Demodex mites are found on the skin of many healthy individuals. Demodex mites in high densities are considered to play a pathogenic role.

**Objective:**

To investigate the association between Demodex infestation and the three most common facial dermatoses: acne vulgaris, rosacea and seborrheic dermatitis.

**Methods:**

This prospective, observational case-control study included 127 patients (43 with acne vulgaris, 43 with rosacea and 41 with seborrheic dermatitis) and 77 healthy controls. The presence of demodicosis was evaluated by standardized skin surface biopsy in both the patient and control groups.

**Results:**

In terms of gender and age, no significant difference was found between the patients and controls (*p* > 0.05). Demodex infestation rates were significantly higher in patients than in controls (*p* = 0.001). Demodex infestation rates were significantly higher in the rosacea group than acne vulgaris and seborrheic dermatitis groups and controls (*p* = 0.001; *p* = 0.024; *p* = 0.001, respectively). Demodex infestation was found to be significantly higher in the acne vulgaris and seborrheic dermatitis groups than in controls (*p* = 0.001 and *p* = 0.001, respectively). No difference was observed between the acne vulgaris and seborrheic dermatitis groups in terms of demodicosis (*p* = 0.294).

**Study limitations:**

Small sample size is a limitation of the study. The lack of an objective scoring system in the diagnosis of Demodex infestation is another limitation.

**Conclusion:**

The findings of the present study emphasize that acne vulgaris, rosacea and seborrheic dermatitis are significantly associated with Demodex infestation. Standardized skin surface biopsy is a practical tool in the determination of Demodex infestation.

## Introduction

Demodex mites were first reported by Jakup Henle in 1871, and detailed descriptions and demonstrations of the pathogen were made in the following years.^1^ The Demodex mite belongs to the family Demodicidae. Demodex folliculorum and Demodex brevis are the two types of Demodex mites that are present on human skin and follicles.^2^ Although the parasite may be found on every area of human skin, the mite has a predilection for the facial area. Demodex mites may be found on normal skin with a density of <5 mites/cm^2^. A diagnosis of demodicosis or Demodex infestation is considered when clinical signs/symptoms appear and when more than 5 mites/cm^2^ are present or when they penetrate into the dermis.^3^, ^4^, ^5^, ^6^

Recently, studies evaluating Demodex infestations have increased. The role of demodicosis has been investigated in some facial conditions/dermatoses, and Demodex mites have been reported to be associated with various skin manifestations, including pityriasis folliculorum,^3^ papulopustular and granulomatous rosacea,^4^, ^5^ pustular folliculitis,^7^ inflammatory papule,^8^ folliculitis,^9^ Seborrheic Dermatitis (SD),^10^ perioral dermatitis^11^ and blepharitis.^12^, ^13^

Rosacea, acne vulgaris (AV) and SD are the three most common inflammatory facial dermatoses; they affect the pilosebaceous unit and have a predilection for the sebaceous gland-rich facial areas.^6^ Demodex mites are also found in the pilosebaceous unit, causing inflammation and leading to immune reactions.^10^ The present study was conducted to investigate the association between AV, rosacea and SD and demodicosis.

## Methods

The study was reviewed and approved by the local ethics committee (protocol no22481095-020-1956, date of approval: 19/09/2018), and all participants gave written informed consent. The study was carried out according to the principles expressed in the Declaration of Helsinki.

A case–control study was planned to investigate the relationship between demodicosis and facial dermatoses, such as AV, rosacea and SD. Patients with only papulopustular AV and only papulopustular rosacea were included. In the SD group, patients with malar area, eyebrows and/or chin or cheek area involvement were enrolled in the study. All diagnoses were made based on clinical examinations by the same dermatologist. Complicated variants of each disease were excluded from the study.

The study included 127 patients (43 with AV, 43 with rosacea and 41 with SD) and 77 healthy controls. The control group comprised 77 healthy people, either medical students or hospital staff, who were matched for age and gender, did not have any disease, and were not receiving any systemic or topical treatment. All participants had Fitzpatrick skin type 2 or 3. For each patient, the age, sex, clinical diagnosis, symptoms, other potential facial dermatoses, recent treatment for the facial condition and date of consultation were recorded. None of the subjects were under any topical treatment, including moisturizers, within the last two months. Subjects with a history of any ablative facial treatments (e.g. peeling and laser) in the prior six months were also excluded. Patients with a history of any systemic disease, systemic treatment within six months of the study and who have smoking habit were also excluded.

**Figure 1:**
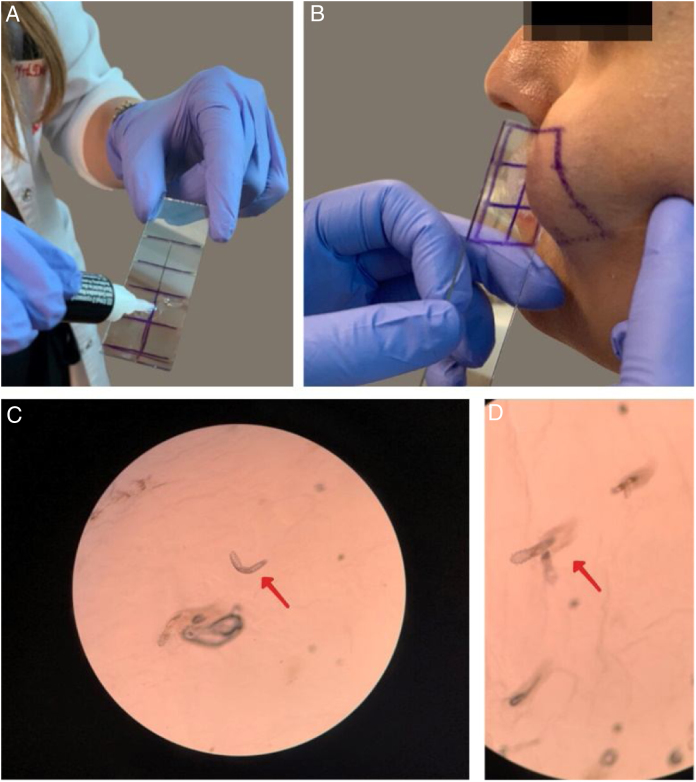
Performing the microscopic examination of mites was performed by cyanoacrylate glue Standardized Skin Surface Biopsy (SSSB). (A) Preparation of the slide covered with cyanoacrylate glue; (B) Collection of the sample from the cheek; (C and D) Microscopic examination of the Demodex mites (×40).

The presence of demodicosis was evaluated by the same dermatologist in each patient. The microscopic examination of mites was performed by cyanoacrylate glue Standardized Skin Surface Biopsy (SSSB) in both the patient and control groups. Two samples were taken for mite examination from the erythematous/inflammatory lesions of the disease on the face. Samples were collected from cheeks and frontal area in controls. A slide covered with cyanoacrylate glue and a marked square was pressed against the skin surface. After 30 s, the slide was removed and the samples were collected. The preparation was examined under a light microscope at 40× and 100× magnification. The performance of the SSSB is shown in fig. 1. The result was considered positive when there were more than five Demodex mites in a 1 cm^2^ area by SSSB.^14^ Most of the Demodex observed by SSSB were D. folliculorum. D. brevis, which mainly lives deeper in the sebaceous glands, is rarely observed with this sampling method.^15^

### Statistical analysis

The Number Cruncher Statistical System 2007 (NCSS; Kaysville, Utah, USA) program was used for the statistical analysis. The descriptive data were expressed with mean ± standard deviation, median, frequency and ratio. In the analysis of normally distributed variables, an independent sample Shapiro–Wilks test was applied to discern the differences between the two groups. The differences between the two independent groups were also examined using the Kruskal Wallis test and Dunn's test for non-normally distributed variables. To compare qualitative variables, the Mann Whitney U test, Pearson × 2 test and Bonferroni corrected × 2 Post hoc test were used. The results were within the 95% Confidential Interval, and *p* < 0.05 was considered statistically significant.

## Results

A total of 204 subjects were enrolled in the study, including 127 patients with facial dermatosis and 77 healthy controls. In terms of gender and age, no significant difference was found between the patients and controls (*p* > 0.05); 66 of the 127 patients (52.0%) had a Demodex infestation, while only two of the 77 healthy controls (2.6%) had infestations. Demodex infestation rates were significantly higher in patients than in controls (*p* = 0.001) (Table 1).

**Table 1:** Demographic data and the presence of Demodex infestation in patients and controls

		Patient group (*n* = 127)	Control group (*n* = 77)	*p*
Age (years)	Min–Max (Median)	15–79 (30)	16–64 (31)	Z: −0.291
	Meant ± SD	32.36 ± 11.61	32.90 ± 11.25	0.771^a^

Gender	Female	82 (61.7%)	51 (38.3%)	*χ*^2^: 0.059
	Male	45 (63.4%)	26 (33.8%)	0.809^b^

Demodex infestation	Absent	61 (48.0%)	75 (97.4%)	*χ*^2^: 52.580
	Present	66 (52.0%)	2 (2.6%)	0.001^b^, ^c^

a Pearson Chi-Square test.

b Mann Whitney *U* test.

c *p* < 0.01.

The patient groups were composed of 43 AV, 43 rosacea and 41 SD patients. The comparisons that were made between these groups and controls are shown in Table 2, Table 3.

**Table 2:** Comparison of demographic data and the presence of Demodex infestation between patients with acne vulgaris, rosacea, seborrheic dermatitis and controls

		Acne vulgaris (*n* = 43)	Rosacea (*n* = 43)	Seborrheic dermatitis (*n* = 41)	Control group (*n* = 77)	*p*
Age (years)	Min–Max (Median)	15–36 (26)	23 − 79 (37)	17–57 (28)	16–64 (31)	*χ*^2^: 41.602
	Mean ± SD	25.49 ± 5.95	40.70 ± 12.83	30.83 ± 9.09	32.90 ± 11.25	0.001^a^, ^c^

Gender	Female	35 (81.4%)	31 (72.1%)	16 (39.0%)	51 (66.2%)	*χ*^2^: 18.287
	Male	8 (18.6%)	12 (27.9%)	25 (61.0%)	26 (33.8%)	0.001^b^, ^c^

Demodicosis	No	31 (72.1%)	9 (20.9%)	21 (51.2%)	75 (97.4%)	*χ*^2^: 78.183
	Yes	12 (27.9%)	34 (79.1%)	20 (48.8%)	2 (2.6%)	0.001^b^, ^c^

a Kruskal Wallis Test.

b Pearson Chi-Square test.

c *p* < 0.01.

**Table 3:** Subgroup comparisons in terms of age, gender and the presence of Demodex infestation

Groups	*p* (age)^a^	*p* (gender)^b^	*p* (Demodex infestation)^b^
Rosacea – AV	0.001^d^	1.000	0.001^d^
Rosacea – SD	0.001^d^	0.012^c^	0.024^c^
Rosacea – Controls	0.001^d^	1.000	0.001^d^
AV – SD	0.151	0.001^d^	0.294
AV – Controls	0.001^d^	0.462	0.001^d^
SD – Controls	1.000	0.024^c^	0.001^d^

AV, acne vulgaris; SD, seborrheic dermatitis.

a Bonferroni Dunn's Test.

b Bonferroni Corrected Pearson Chi-Square test.

c *p* < 0.05.

d *p* < 0.01.

The mean age of patients with rosacea was significantly higher than for the AV patients, SD patients and controls (*p* = 0.001, *p* = 0.001, and *p* = 0.001, respectively). The mean age of AV patients was significantly lower than the controls (*p* = 0.003).

In terms of gender, the male-to-female ratio was significantly higher in the SD group compared with the AV, SD and control groups (*p* = 0.012, *p* = 0.001, and *p* = 0.024; respectively).

Demodex infestation rates were significantly higher in the rosacea group than in the AV, SD groups and controls (*p* = 0.001, *p* = 0.024, and *p* = 0.001, respectively). Demodex infestation was found to be significantly higher in the AV and SD groups than in the controls (*p* = 0.001, *p* = 0.001, respectively). No difference was seen between the AV and SD groups in terms of demodicosis (*p* = 0.294).

## Discussion

This study demonstrated that Demodex infestation was associated with AV, rosacea and SD. The highest incidence of infestation was observed in the rosacea patients, followed by SD and AV. The presence of Demodex infestation was significantly higher in the rosacea patients than in the AV and SD patients and controls, whereas the infestation was significantly more common in the AV and SD patients as compared with controls.

D. folliculorum and D. brevis, the two Demodex species, are ubiquitously found on the normal skin of adult humans, particularly in the pilosebaceous units of the face.^16^ D. folliculorum resides within the hair follicle, whereas D. brevis is found predominantly in the sebaceous and meibomian glands.^2^ Demodex mites penetrate into skin cells (particularly the keratinocytes that line pilosebaceous follicles) and ingest their contents. Demodex mites feed on the sebum and cellular proteins that are obtained by protease containing the salivary enzymes of the mites.^16^, ^17^ The lipase enzymes of Demodex are also thought to play a role in digesting bacteria or other microorganisms in addition to the digestion of lipid material.^18^, ^19^ The enzymatic process leads to degradation of the follicular epithelium, which may result in perifollicular inflammation.^16^, ^17^ Demodex mites may also cause mechanical blockage of the follicle opening. Moreover, it is thought that extrafollicular mites may induce a granulomatous foreign body reaction through their chitinous exoskeleton. Dying mites are thought to trigger an immune response in the host by releasing their internal contents and the chitinous exoskeletons of degrading, dying mites, followed by inflammatory changes.^20^, ^21^, ^22^ Demodex mites may also suppress the innate immune response of the hosts that provide for their survival.^16^ It has been shown that the Tn antigen, which is a carbohydrate coating providing protection for cancer cells and parasites from immunity, is expressed by Demodex mites.^23^, ^24^ Demodex mites are also shown to affect the secretion of inflammatory cytokines, such as IL-8 and TNF-alpha and TLR expression, through the interaction with cells of the pilosebaceous unit.^23^, ^24^, ^25^, ^26^, ^27^, ^28^

The cause of clinical findings in Demodex infestation is still unknown; but the aforementioned mechanisms are considered to play a role in the occurrence of Demodex infestation. Although demodicosis has been demonstrated in several skin conditions, the role of Demodex mites in dermatologic conditions is still controversial.

AV is a multifactorial disease of the pilosebaceous unit. It can be classified as comedonal, papulopustular and nodular acne. Although the etiology of AV remains unclarified, androgen, increasing sebum secretion, hyperkeratosis of the pilosebaceous duct, follicular orifice blockage and the proliferation of propionibacterium acne are some of the factors thought to contribute in the development of AV.^29^, ^30^ Demodex mites may contribute to the development of acne lesions through follicle blockage, leading to distension and intrafollicular hyperkeratosis and causing inflammation and immune reactions.^31^, ^32^

Recently, many studies evaluating the relationship between AV and Demodex infestation have been conducted. Reportedly, 11.8% of 101 AV patients^33^ and 15.38% of 78 AV patients^34^ showed Demodex positivity in seperate studies. In another, no significant association between Demodex and AV was observed.^35^ In a recent meta-analysis, 48 of 63 articles demonstrated a positive association between Demodex infestation and AV, while 15 showed controversial findings.^31^ In our study, significantly higher Demodex positivity was observed in AV patients than in healthy controls.

Rosacea is a common chronic inflammatory cutaneous disorder, mostly seen over the age of 30 years, with remission and exacerbation periods.^36^, ^37^ Flushing, erythema, telangiectasia, edema, papules, pustules, phymatous changes and ocular lesions are the signs of the disease.^36^, ^37^, ^38^ Rosacea is classified as Erythematotelangiectatic Rosacea (ETR), Papulopustular Rosacea (PPR), phymatous rosacea, ocular rosacea and variant granulomatous rosacea according to the American National Rosacea Society (NRS) Expert Committee classification and staging system, which also acts as a diagnostic tool.^36^, ^37^ Various factors, including abnormalities in cutaneous vascular homeostasis, climatic exposures, dermal matrix degradation, chemical and ingested agents, pilosebaceous unit abnormalities and microbial organisms, have been introduced in the pathogenesis, though the etiology of rosacea remains uncertain.^37^, ^38^ Recently, many studies have been conducted examining the relationship between Demodex mites and rosacea, mostly demonstrating a positive relationship. Demodex mites may contribute to the pathogenesis of rosacea in several ways. The blockage of hair follicles and sebaceous glands by an increased number of mites may result in cutaneous barrier disruption and tissue damage. Subsequent increases in TLR expression, chitin mite exoskeletons and releases of internal mite contents, including bacterial antigens, may trigger an inflammatory reaction and also result in an immune response followed by neutrophil and macrophage activation.^26^, ^27^, ^39^, ^40^, ^41^ Particularly, rosacea T-cell-mediated immune responses to Demodex have been reported to play a role in the pathogenesis. Predominantly CD4 helper/inducer T lymphocytes have been demonstrated in granulomas and in perifollicular infiltrates.^5^, ^42^, ^43^, ^44^ Moreover, humoral immunity has also been suggested to play a role in inflammatory reactions.^45^

Clinical studies have revealed increased numbers of Demodex mites on the skin of rosacea patients compared with healthy controls.^45^ Demodicosis has been reported to be higher on the cheeks of patients with PPR than on controls with healthy skin.^4^, ^5^, ^45^, ^46^ In the present study, 34 of the 43 rosacea patients had Demodex infestations, which was significantly higher than AV and SD patients and healthy controls.

SD, a chronic and superficial inflammatory dermatosis of the skin, is characterized by erythematous, oily yellow squames on the sebaceous gland-rich areas of the skin, including the scalp, face, chest, back and flexural areas.^47^, ^48^ Increased sebum activity, Pityrosporum ovale infection, drugs, immunological abnormalities, genetic predisposition, neurological disorders, emotional stress, diet, lifestyle and environmental factors have been identified as contributors in the pathogenesis of the disease or aggravating SD symptoms; still, the exact etiology of SD remains unknown.^10^, ^49^ Demodex mites tend to be found on the predilection areas of the SD. We believe that Demodex-induced inflammation may also contribute in the pathogenesis of SD. Karincaoglu et al.^10^ demonstrated significantly higher Demodex positivity on the lesional and non-lesional skin of SD patients than healthy controls. They also suggested that SD itself may be a predisposing factor to Demodex infestation, but no data supporting this hypothesis are available.^10^ In our study, we also demonstrated a higher presence of Demodex infestation in SD patients than in controls.

The study has a few limitations. For example, we only made the diagnosis of Demodex infestation without an objective scoring system, which limits making comments on the association between the severity of the infestation and the dermatoses. D. folliculorum is the most demonstrated mite by SSSB, as it resides within the follicles, while D. brevis lives deeper. The absence of an examination of D. brevis, which may also contribute in the pathogenesis of the diseases, is a limitation of the study. The small sample size is another limitation.

## Conclusion

It is still unclarified whether demodicosis is the cause of skin diseases. But based on the findings of the present study, it may be concluded that, rosacea, AV and SD are significantly associated with Demodex infestation. The reactivation of the immune system, inflammation and follicular changes caused by the Demodex mites might contribute in the development of the diseases. SSSB, an easily accessible and practical tool, may be used to determine the presence of a Demodex infestation. Particularly in cases resistant to therapies, an accompanying Demodex infestation should be considered.

## Financial support

None declared.

## Authors’ contributions

Ezgi Aktaş Karabay: Approval of the final version of the manuscript; conception and planning of the study; elaboration and writing of the manuscript; obtaining, analysis, and interpretation of the data; effective participation in research orientation; intellectual participation in the propaedeutic and/or therapeutic conduct of the studied cases; critical review of the literature; critical review of the manuscript.

Aslı Aksu Çerman: Statistic analysis; approval of the final version of the manuscript; critical review of the manuscript.

## Conflicts of interest

None declared.
